# Advances in synthetic strategies for two-dimensional conjugated polymers

**DOI:** 10.1039/d4qo02211d

**Published:** 2025-01-28

**Authors:** Ruyan Zhao, Guoqin Liu, Philomène Leonore Koko, Mingchao Wang, Xinliang Feng

**Affiliations:** a Max Planck Institute of Microstructure Physics Weinberg 2 06120 Halle Germany; b Center for Advanced Electronics Dresden (cfaed) & Faculty of Chemistry and Food Chemistry, Technische Universität Dresden Mommsenstraße 4 01062 Dresden Germany xinliang.feng@tu-dresden.de; c School of Advanced Materials, Peking University, Shenzhen Graduate School Shenzhen 518055 China mingchao.wang@pku.edu.cn

## Abstract

Two-dimensional conjugated polymers (2D CPs) are typically represented by 2D conjugated covalent organic frameworks (COFs) that consist of covalently cross-linked linear conjugated polymers, which possess extended in-plane π-conjugation and out-of-plane electronic couplings. The precise incorporation of molecular building blocks into ordered polymer frameworks through (semi)reversible 2D polycondensation methodologies enables the synthesis of novel polymer semiconductors with designable and predictable properties for various (opto)electronic, spintronic, photocatalytic, and electrochemical applications. Linkage chemistry lays the foundation for this class of synthetic materials and provides a library for subsequent investigations. In this review, we summarize recent advances in synthetic strategies for 2D CPs. By exploring synthetic approaches and the intricate interplay between chemical structure, the efficiency of 2D conjugation, and related physicochemical properties, we are expected to guide readers with a general background in synthetic chemistry and those actively involved in electronic device research. Furthermore, the discussion will appeal to researchers intrigued by the prospect of uncovering novel physical phenomena or mechanisms inherent in these emerging polymer semiconductors. Finally, future research directions and perspectives of highly crystalline and processable 2D CPs for electronics and other cutting-edge fields are discussed.

## Abstract 2

This review covers synthetic approaches towards crystalline 2D conjugated polymers linked by imines, pyrazines, vinylenes, C–C, or imidazoles. It explores the interplay between structures and properties, highlighting their prospects in electronics.

## Graphical abstract

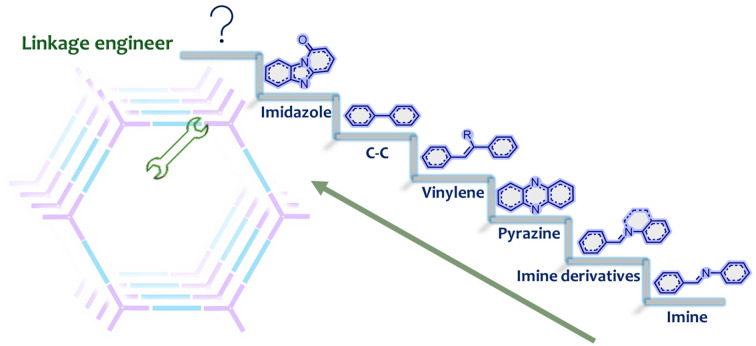

**10th anniversary statement**

It is a great honor to contribute to the 10th-anniversary collection of *Organic Chemistry Frontiers*. Over the past decade, the journal has established itself as a highly visible platform in the fields of organic chemistry and organic materials, fostering innovation and collaboration in the global chemistry community. Our experiences with the journal have been exceptional, marked by rigorous peer review and impactful publication processes. Coincidentally, 2024 also marks the 10th anniversary of our research group at TU Dresden, we have been dedicated to advancing polymer synthesis, supramolecular chemistry, synthetic 2D materials, electronic and energy technologies. Looking ahead, we share the journal's mission to push the boundaries of chemical research and drive transformative discoveries in organic chemistry and advanced organic materials. As the journal celebrates this milestone, we would like to express our wholehearted support for its commitment to advancing chemistry in China, Asia, and beyond, and to inspiring researchers to address pressing scientific challenges. We are confident that the journal will continue to grow and flourish in the years to come.

## 1. Introduction

Conjugated polymers (CPs) are a class of organic semiconductors that feature tunable energy levels and delocalized π-electron clouds along their conjugated chains.^1^ Traditional conjugated polymers usually comprise one-dimensional (1D) backbones decorated with flexible side chains that can be easily integrated into electronic devices through solution-processing methods.^2,3^ Nonetheless, the charge transport performance achieved so far in 1D CPs are still inadequate for high-performance organic electronics: the charge carriers are delocalized only along the linear polymer backbone, and the hopping between adjacent polymer chains limits the charge transport in space. By extending the dimensionality, multiple strands of charge transport can be, in principle, established to bypass defects, narrow the band gaps, and suppress vibration to facilitate band-like transport in the resulting two-dimensional (2D) CPs.^4^

2D CPs are generally represented by 2D conjugated covalent organic frameworks (2D c-COFs),^5^ which possess in-plane extended π-conjugation and out-of-plane electronic coupling.^6^ 2D CPs are assembled from aromatic building blocks linked together by conjugated covalent linkages that can be formed through various 2D polycondensation/polymerization reactions. Highly planar and conjugated building blocks can facilitate the crystallization process and improve in-plane π-delocalization as well as out-of-plane π-orbital overlapping. Linkage chemistry determines the reversibility of the error-correction crystallization and influences the stability and electronic communication between different building blocks. The 2D lattice and the interlayer stacking mode define band structure features, such as Dirac points and topological flat bands.^7^ Researchers should consider the above four important aspects before developing 2D CPs, *i.e.*, suitable building blocks, linkages, lattice, and interlayer stacking modes. In particular, linkage chemistry is essential for the controllable synthesis of crystalline 2D CPs and strongly impacts the underlying physicochemical properties. In addition, the layer numbers (*i.e.*, thickness in the axial direction), the rational aspect ratios, and the periodic alignments are also important merits for 2D CPs.

Unlike 1D CPs that can be prepared efficiently through irreversible transition metal-catalyzed cross-coupling reactions, such as Suzuki/Stille/Sonagashira/Yamamoto polymerization, direct arylation polycondensation (DArP), and Kumada catalyst transfer polycondensation (KCTP),^8–11^*etc*., the synthesis of 2D CPs relies on the reversible or quasi-reversible linkage formation and/or the subsequent error-correction.^12^ Currently, only very limited synthetic methodologies are accessible for the synthesis of crystalline 2D CPs. Among them, 2D polyimines (2D PIs, or imine-linked 2D c-COFs) synthesized through reversible Schiff-base chemistry are the most prevalent. However, the imine-linked 2D c-COFs suffer from low chemical stability and poor π-electron delocalization, making them less attractive for future electronic and spintronic device integrations.^13,14^ The first imine-linked COF was reported in 2009,^13^ while the first 2D PI was developed in 2011.^15^ Subsequently, researchers have made significant efforts to the development of 2D PI derivatives, employing post-synthetic modification of 2D PIs and multiple-component one-pot synthesis (to create *e.g.*, thiazole/pyridine rings) through imine-linked intermediates. These strategies have been employed to enhance the chemical stability and/or conjugation of the resulting materials. Using a similar Schiff-base reaction of α-diketones with α-diamines, ladder-type 2D polypyrazines (2D PPZs, or pyrazine/phenazene-linked 2D c-COFs) can be developed with enhanced framework planarity and π-conjugation.

As early as 2009, aryl–aryl coupling on flat metal surfaces overcame the irreversibility of the C–C coupling reaction by using halide-functionalized monomers, thereby enabling the synthesis of 2D polyarylenes (2D PAs).^16^ For example, a 2D polyphenylene was successfully synthesized on the Ag (111) surface.^17^ However, the obtained thin layer showed limited lateral size (*ca.* 50 nm) and strong binding with the Ag(111) surface, which presents a significant challenge in terms of transferring the thin layer for device integration. In 2016, 2D poly(arylene vinylene)s (2D PAVs, or vinylene/sp^2^-C-linked 2D c-COFs) were synthesized by the Knoevenagel polycondensation in solution, showing much strengthened 2D conjugation and chemical stability compared to 2D PIs.^18^ In comparison to the single-double bond alternation in the above 2D CPs, ladder-type 2D poly(benzimidazobenzophenanthroline)s (2D BBLs or BBL-type 2D c-COFs) represent unique fused molecular geometry to further increase the effective π-conjugation length by strengthened parallel p-orbital interactions. A phthalocyanine-based 2D BBL has exhibited intrinsic charge carrier mobility as high as *ca.* 1000 cm^2^ V^−1^ s^−1^,^4^ showing the potential of 2D CPs in high-performance (opto)electronic and electrochemical applications.

Over the past decade, the growing interest in 2D CPs has prompted significant research efforts to advance their design, synthesis, and potential applications. It is beyond question that the synthesis methodologies provide the foundation and material library for subsequent investigations. To date, no representative review article has provided the progress on the synthesis of 2D CPs. Given our expertise in the chemistry and physics of 2D CPs, we anticipate presenting an overview of the latest developments in this field and offering insights into its prospects. This review aims to provide a comprehensive overview of the strategies employed to synthesize crystalline 2D CPs, including 2D PIs and derivatives, 2D PAs, 2D PAVs, and 2D BBLs. By examining the various synthetic approaches and the intricate relationship between structure and properties, we aim to present a comprehensive guideline for readers with a general background in synthetic chemistry and those engaged in electronic device research.

## 2. Synthetic strategy for 2D CPs

Various approaches, including solution synthesis, on-surface synthesis, and interfacial synthesis, have been explored to synthesize 2D CPs in bulk powders and thin films, with a few exceptions being single crystals. In consideration of the various polymerization and crystallization mechanisms – that is to say, thermodynamic and/or kinetic control – employed in different synthetic strategies, the present review does not seek to provide a comparison of these mechanisms. Solid-state synthesis, combined with exfoliation techniques, has been a method of choice for creating a range of 2D CPs.^19,20^ Nonetheless, this technique requires the meticulous pre-assembly of monomers into single crystals before polymerization, which limits its broad applicability in the synthesis of 2D CPs. In contrast, solution-based synthesis methods, such as hydrothermal or solvothermal processes, stand out as the most efficient and straightforward way of producing 2D CPs in polycrystalline bulk forms. Furthermore, on-surface synthesis and interfacial polymerization are adept at generating thin films of these materials.^21^

### 2.1 Solution synthesis

Solution synthesis, including hydro- and solvothermal synthesis, provides a high degree of adaptability in reaction parameters, encompassing a spectrum of variables like solvent, catalyst, temperature, volume, pressure, *etc*.^22–24^ This adaptable approach relies on the general reversibility or thermodynamic control of reactions, offering benefits such as high selectivity, precise control over stereochemistry, and high yields. It has been commonly used to produce bulk polycrystalline 2D CPs and, in some instances, small-sized single crystals with domain size of *ca.* 1 μm. For the fabrication of thin films, solution processing of exfoliated 2D CPs is essential to maximize their potential across a variety of applications and to explore their layer-dependent physical characteristics.

### 2.2 On-surface synthesis

On-surface synthesis under vacuum conditions is appealing for the meticulous crafting of monolayer 2D CP with tunable lateral size.^25,26^ In this respect, the metal substrates provide confining surfaces and catalytic sites. So far, this method has been limited to 2D PAs synthesized *via* Ullmann-type coupling. The chemical vapor deposition (CVD) technique has proven effective in producing thin films with smooth surfaces and adjustable thicknesses.^27–29^ However, the interaction between molecules and the substrate, along with the high sublimation temperatures of monomers, can restrict their surface mobility, leading to limited crystallinity with domain sizes typically below 100 nm. An additional significant challenge is the tricky transfer process of these thin layers from the initial solid surface to alternative substrates for further characterization and integration into devices.

### 2.3 Interfacial synthesis

The interface between two immiscible liquids, such as chloroform/water or dichloromethane/water, forms a nanoscale-thick boundary that is inherently uneven due to the minor solubility of one liquid in the other.^30^ This characteristic makes liquid/liquid interfacial synthesis an effective technique for creating multilayer or relatively thick films spanning from a few nanometers to micrometers.^31^ Techniques like on-water surface synthesis, such as surfactant-monolayer-assisted interfacial synthesis (SMAIS), provide a smooth and well-defined surface with a root mean square roughness of approximately 3 Å,^32^ which is ideal for the large-scale fabrication of monolayer to few-layer 2D CPs.^21,33–35^ These approaches also simplify the transfer of the synthesized films from the water surface onto various substrates for subsequent characterization and device fabrication. Recent studies utilizing these methods have produced highly crystalline 2D PIs,^36,37^ 2D poly(pyridinium salt),^38^ 2D PAVs,^39^*etc*., with adjustable thicknesses from ∼1 to ∼200 nm.

## 3. Linkage chemistry towards crystalline 2D CPs

The choice of linkage is a pivotal factor for the construction of 2D CPs, as they affect the stability and the degree of conjugation, which in turn dictates the physicochemical properties of the 2D CPs.^12^ Typical conjugated linkages discussed in this Review include imine (formed by Schiff-base condensation between aromatic aldehyde and amine, Table 1, Fig. 2 and 3), pyrazine (prepared by two consecutive Schiff-base reactions of α-diketone with α-diamine), vinylene (furnished by the different condensation reaction of aromatic aldehyde and active methyl, *i.e.* Knoevenagel/Aldol-type/Horner–Wadsworth–Emmons (HWE)/Wittig reaction), C–C (formed mainly by Ullmann-type coupling), and BBL-imidazole (prepared by imide bond formation between α-diamine and naphthalene anhydride followed by intramolecular dehydration).^4,40^ Imine linkages are commonly utilized because of their facile formation of highly crystalline 2D CPs. Yet, they are characterized by moderate π-electron delocalization due to the inherent polarity of the imine (C=N) bonds. The ring structure of pyrazine linkage enhances conjugation and chemical stability and facilitates charge carrier transport. Compared to 2D PIs and PPZs, 2D PAVs, 2D PAs, and 2D BBLs are difficult to synthesize due to the challenges associated with low reversibility of C=C bond/BBL-imidazole formation or irreversibility of C–C bond formation. From the perspective of enhancing the in-plane conjugation, we will discuss the 2D PIs, 2D PPZs, 2D PAVs, 2D PAs, and 2D BBLs in sequence.

Post-modification of 2D PIs to construct conjugated linkages with better chemical stability and/or enhanced conjugation

**Table:** 

Schiff-base reaction 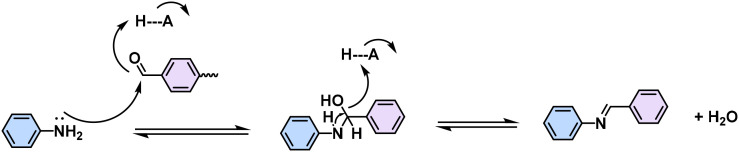	
Transformation of imine to other aromatic linkages	Ref.
Imine to substituted and unsubstituted-quinoline 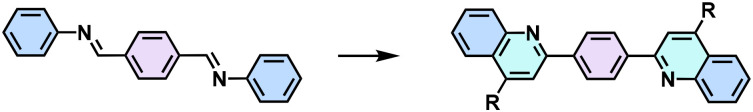	58
Imine to oxazole or thiazole 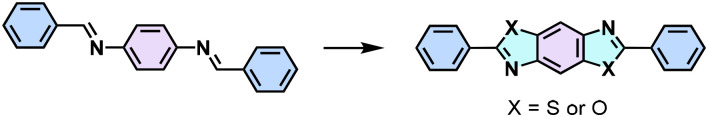	59
Imine to thieno[3,2-*c*]pyridine 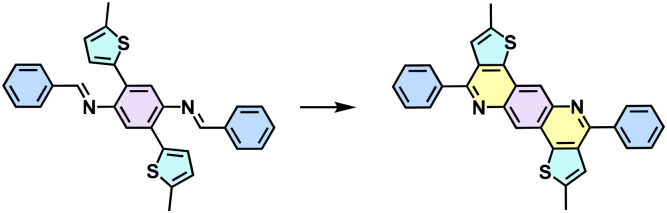	60

**Table:** 

Multicomponent one-pot synthesis through imine-linked intermediates	Ref.
Thiazole (C–H functionalization and oxidative annulation) 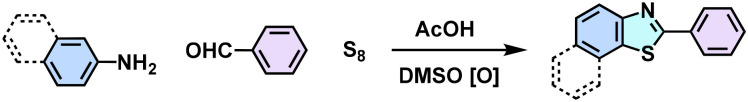	61
Benzoxazole (imine formation, cyclization, and oxidation) 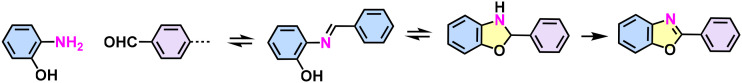	64
Non-substituted quinoline 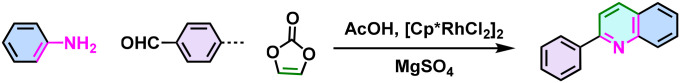	65
Substituted quinoline (Povarov reaction) 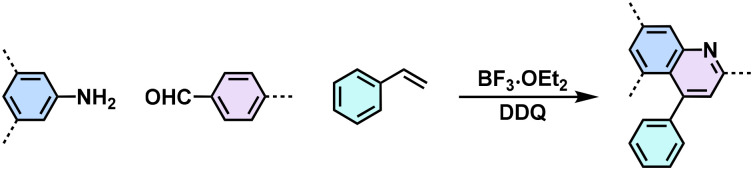	66
Pyrimidazole (Groebke–Blackburn–Bienaymé reaction) 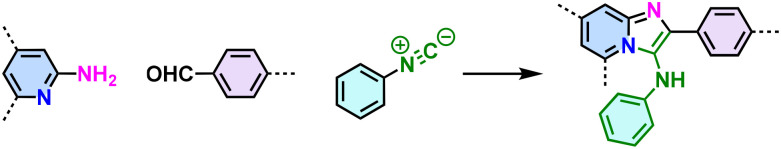	67

### 3.1 2D polyimines (2D PIs) by Schiff-base reaction

**Scheme 1:**
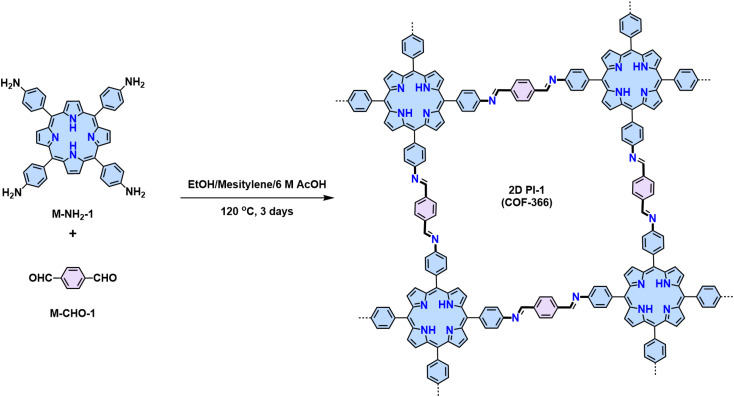
Synthesis of the first 2D polyimines of 2D PI-1 (COF-366).

Since the pioneering work of 2D PIs (COF-366, named as 2D PI-1 in this Review) between terephthalaldehyde–4,4′,4′′,4′′′-(porphyrin-5,10,15,20-tetrayl)tetraaniline (M-NH_2_-1, the monomers are also renamed in this review: M means monomer followed by its functional groups such as –NH_2_, –CHO, –CH_3_, –CH_2_CN, *etc*.) and terephthalaldehyde (M-CHO-1) by Yaghi and colleagues in 2011 (Scheme 1),^15^ many efforts, from both synthetic and application aspects, have been made to achieve high-crystalline 2D PIs and the corresponding electronic device integrations.^12,41–43^ Structural modulation according to the experimental powder X-ray diffraction (pXRD) pattern reveals that 2D PI-1 adopts a fully eclipsed structure with an AA stacking sequence, exhibiting an interlayer distance of 5.64 Å.^15^ The formation of imine linkage includes two steps. First, the amine nitrogen acts as a nucleophile, attacking the electrophilic carbonyl carbon of aldehydes. Then, the nitrogen is deprotonated, and the electrons from this N–H bond push the oxygen off the carbon, leaving a compound with a C=N double bond and releasing a water molecule (Table 1). Because of the highly reversible reaction merit, crystalline 2D PIs are experimentally the most feasible. During the past decade, various 2D PIs have been developed with robust topologies like honeycomb, rhombic, square, Kagome, hexagonal, *etc*. Both polycrystalline bulk powders, single crystals, and wafer-size thin films have been synthesized.^44–50^ For comprehensive discussions about the imine-linked 2D PIs, we recommend readers turn to recent comprehensive reviews.^12,41,51^

**Fig. 1:**
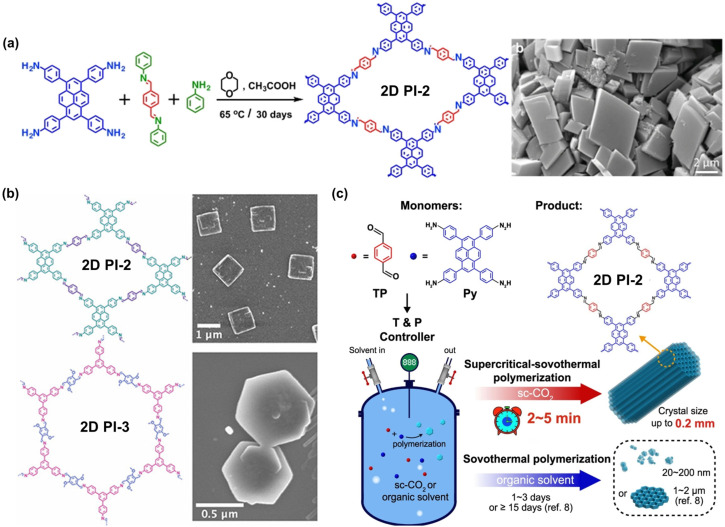
Representative 2D CP single crystals. (a) Planar building block gives easy single crystal synthesis of 2D PI-2 (ref. 45). (b) The synthesis of 2D PI-2 and 2D PI-3 single crystals at ambient pressure and short reaction times (ref. 49). Adapted with permission American Chemical Society. (c) Schematic of the 2D PI-2 single crystals in supercritical CO_2_ (ref. 44).

The growth of single crystals of 2D CPs can facilitate a deeper understanding of the 2D polymer crystallization and growth process at the atomic level. This will provide more accurate information about the structure–property relationship, thus guiding the design of the new generation of electronic materials.^52^ The high reversibility of imine-bond formation and deformation allows for achieving 2D single crystals by delicately tuning the nucleation and growth dynamics. Zhao *et al.* disclosed that building blocks with a large π-conjugated planar structure, *e.g.*, 4,4′,4′′,4′′′-(pyrene-1,3,6,8-tetrayl)tetraaniline (M-NH_2_-3), can facilitate the self-assembly and pre-arrangement of monomers *via* enhanced intermolecular interactions, which is beneficial to the growth of single crystals (Fig. 1a).^45^ Dichtel *et al.* synthesized two single crystals of 2D PI-2 and 2D PI-3 using benzoic acid as the catalyst and aniline as a monofunctional modulator in benzonitrile (Fig. 1b).^49^ Instead of crystallizing in a typical organic solvent, Wei and colleagues used supercritical CO_2_ (sc-CO_2_) as the solvent medium to accelerate single-crystal polymerization by 10 000 000 folds and produce 2D PI-2 single crystals with sizes up to 0.2 mm within 2–5 min (Fig. 1c).^44^ Cortés and colleagues used optical interferometric scattering microscopy for the operando studies of COF polymerization and framework formation.^52^ It is noteworthy that the size and quality of 2D PI crystals achieved so far are still not comparable to those of 3D PI crystals, due to strong π-π stacking of planar building blocks and thus low solubility of crystal seeds in solvent. A structure resolution by single-crystal X-ray diffraction remains a dream for 2D PIs.^47^ A more profound comprehension of growth mechanisms is imperative to direct the synthesis of high-quality 2D PI single crystals.

**Fig. 2:**
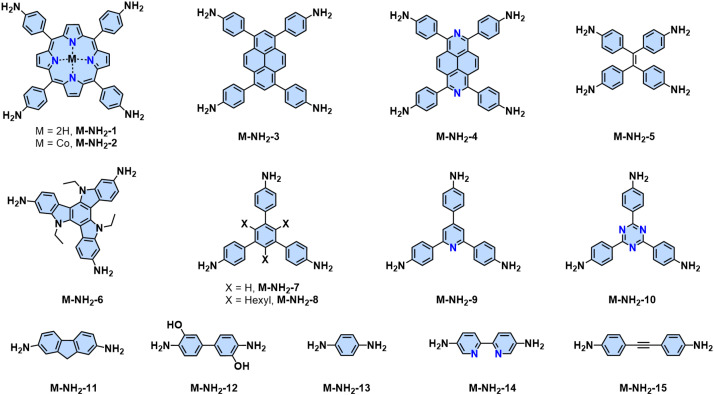
Representative monomers contain –NH_2_ groups for constructing 2D PIs.

**Fig. 3:**
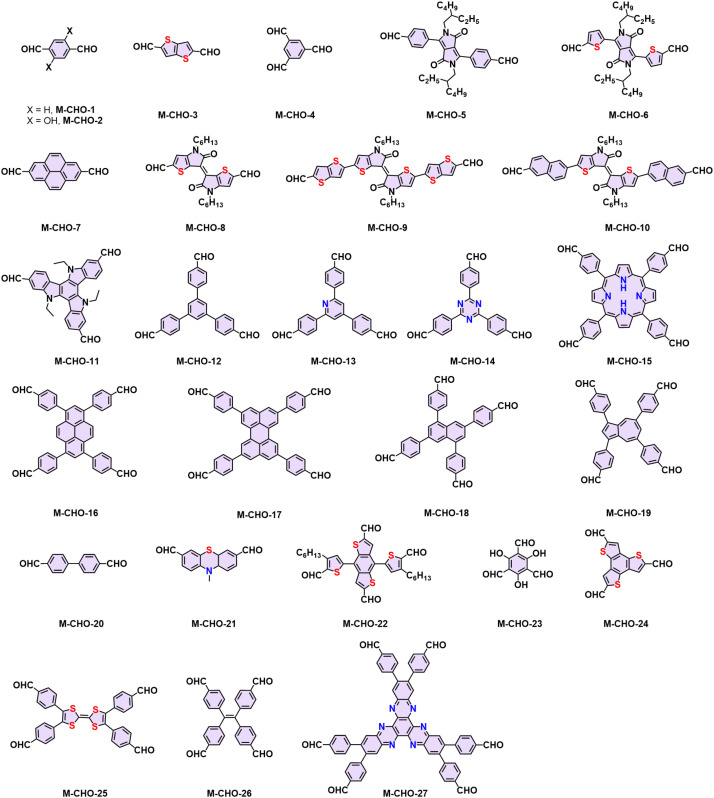
Representative aromatic aldehydes for constructing 2D PIs and 2D PAVs.

2D PI films have been synthesized using on-surface and interfacial synthesis methods. Our group prepared porphyrin-containing monolayer and multilayer 2D CPs from M-NH_2_-1/2 and 2,5-dihydroxyterephthalaldehyde (M-CHO-2) at an air/water and liquid/liquid interface, respectively.^53^ Both the monolayer and multilayer 2D CPs have crystalline structures as indicated by selected area electron diffraction. The monolayer 2D CP has a thickness of ∼0.7 nm with a lateral size of 4-inch wafer and a Young's modulus of 267 ± 30 GPa. The field-effect transistor fabricated from this 2D CP shows a charge mobility of 1.3 × 10^−6^ cm^2^ V^−1^ s^−1^, which can be increased over 100 times after doping with iodine. Later, various highly crystalline and large-area 2D PIs were developed *via* the SMAIS method, showing tunable thicknesses (1 to 100 nm) and large crystalline domain sizes (up to 120 μm^2^).^50,54–57^

### 3.2 2D PI derivatives by post-modification or one-pot synthesis

The high reversibility of imine linkages would conversely endow poor π-electron delocalization due to the polarized C=N bonds and poor stability against strong acids/bases. Consequently, different post-synthetic methods have been developed to enhance the stability and the conjugation of 2D PIs or imine-linked intermediates (Table 1). For example, Liu and colleagues reported a facile and general strategy to transform imine-linked 2D PIs into a series of quinoline-linked 2D CPs utilizing an aza-Diels–Alder cycloaddition reaction, reacting with various aryl alkynes.^58^ Yaghi and coworkers converted imine-linked 2D PIs based on 1,4-phenylenediamine (M-NH_2_-13) and 1,3,6,8-tetrakis(4-formylphenyl)pyrene (M-CHO-16) through consecutive linker substitution and oxidative cyclization to two isostructural 2D CPs, having thiazole and oxazole linkages.^59^ Zhao and colleagues next reported an approach for constructing thieno[3,2-*c*]pyridine-linked 2D CPs, featuring a combination of imine formation and a post-oxidative cyclization.^60^ These resultant 2D CPs generally exhibit enhanced chemical stability and have enabled them to visualize the periodic structural characteristics as honeycomb-like porous structures by high-resolution TEM (HR-TEM) after transformation. However, the corresponding 2D PI counterparts are extremely unstable under the electron beam, and HR-TEM images could not be obtained so far.

Despite the enhanced conjugation, the post-modification approach frequently results in framework collapse and reduced crystallinity during solid-state transformations, which ultimately compromises the physicochemical properties of 2D CPs. As an alternative approach, multicomponent one-pot or *de novo* synthesis *via* imine intermediates has the potential to yield crystalline 2D CPs from the outset, thereby circumventing the inefficiencies associated with solid-state post-modification. This method can also create 2D CPs that are otherwise unattainable through post-modification.^61–63^ Cooper and his coworkers developed a simple and efficient three-component assembly reaction between readily available aldehydes, amines, and elemental sulfur *via* a C–H functionalization and oxidative annulation under transition-metal-free conditions.^61^ Wang proposed cascade reactions to construct stable and crystalline benzoxazole-linked 2D CPs.^64^ Xiang and coworkers prepared non-substituted quinoline-linked 2D CPs through post-synthetic modification strategies and one-pot synthesis methods.^65^ The mechanism for the construction of non-substituted quinoline linkages starts with the formation of an imine through the condensation of aldehyde and amine, followed by Rh-catalyzed [4 + 2] annulation between the *in situ* generated imine and vinylene carbonate. In addition, Dong and coworkers successfully synthesized phenyl-substituted quinoline-linked 2D CPs through one-pot aza-Diels–Alder cycloaddition (Povarov) reactions under solvothermal conditions in high yields.^66^ Wang and coworkers utilized the representative Groebke–Blackburn–Bienaymé (GBB) reaction based on isocyanide chemistry to construct a series of pyrimidazole-based COFs in one step from isocyanide, aminopyridine, and aldehyde monomers.^67^ The formation of fused imidazole rings within the frameworks is ubiquitous, ensuring excellent chemical stability (Table 1).

### 3.3 2D polypyrazines (2D PPZs) by Schiff-base reaction

**Scheme 2:**
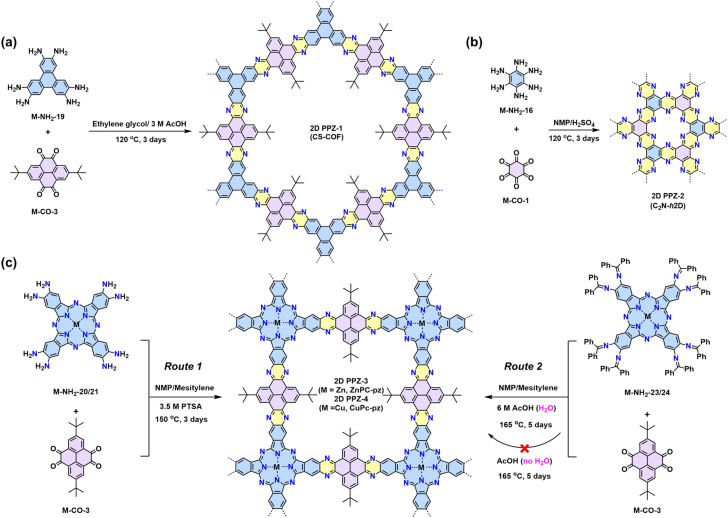
(a) Synthesis of the first crystalline pyrazine-bridged 2D polypyrazine of 2D PPZ-1 (CS-COF). (b) Synthesis of 2D PPZ-2 crystals. (c) Two routes to synthesize 2D PPZ-3 (ZnPc-pz) and 2D PPZ-4 (CuPc-pz) with cubic lattice.

**Fig. 4:**
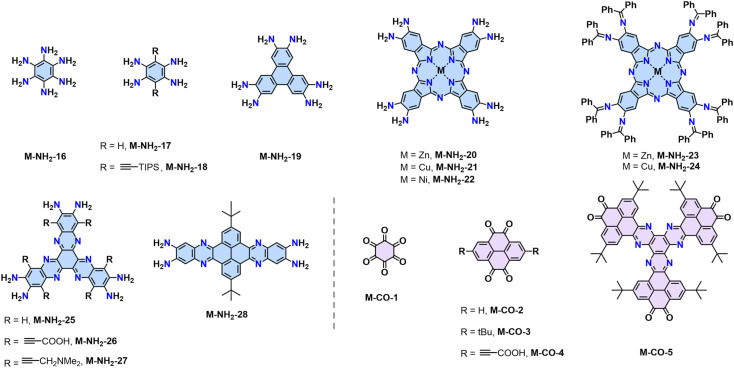
Representative monomers contain multiple –NH_2_ (blue) and –C=O (pink) groups at the -*ortho* positions to construct 2D PPZs.

The Schiff-base reaction mechanism is also employed for the synthesis of 2D PPZs, utilizing monomers with multiple amine groups and ketone (–CO) groups at the -*ortho* position.^68,69^ In 2013, Jiang reported a triphenylene-based 2D PPZs (CS-COF, designated as 2D PPZ-1 in this review) that exhibits a fully fused backbone and semiconducting character (Scheme 2a).^68^ Later, Baek and coworkers reported the synthesis of 2D PPZ-2 (C_2_N-*h*2D) crystals through the condensation of benzene-1,2,3,4,5,6-hexaamine (M-NH_2_-19) and cyclohexane-1,2,3,4,5,6-hexaone (M-CO-1) (Scheme 2b).^69^ Other amino monomers including triphenylene-2,3,6,7,10,11-hexaamine (M-NH_2_-19), 2,3,9,10,16,17,23,24-octaaminophthalocyaninato Zn/Cu(ii) (M-NH_2_-20/21), and ketone monomers including 2,7-di-*tert*-butylpyrene-4,5,9,10-tetraone (M-CO-3) and 2,7,12,17,22,27-hexa-*tert*-butylphenanthro[4,5-*abc*]phenanthro[4′,5′:5,6,7,8]quinoxalino[2,3-*h*]phenanthro[4′,5′:5,6,7,8]-quinoxalino[2,3-*j*]phenazine-9,10,19,20,29,30-hexaone (M-CO-5), were also developed for the synthesis of 2D PPZs (Fig. 4).

In 2019, our group demonstrated two metal-phthalocyanine-based 2D PPZs, namely ZnPc-pz COF and CuPc-pz COF (2D PPZ-3 and 2D PPZ-4), *via* the direct Schiff-base condensation between M-NH_2_-20/21 and M-CO-3. pXRD analysis reveals the crystalline nature of both 2D PPZs with *a* = *b* = 22.2 Å and an interplane distance of *ca.* 3.30 Å for a serrated AA stacking mode.^70^2D PPZ-3 and 2D PPZ-4 are p-type semiconductors with an optical band gap of ∼1.2 eV and charge mobility of ∼5 cm^2^ V^−1^ s^−1^.^70–72^ Reversible p-doping of the materials with iodine can enhance not only the electrical conductivity but also the charge carrier scattering time and mobility of 2D PPZs.^73^ Additionally, they can also be synthesized through a multistep Schiff-base reaction from the –N=CPh_2_ protected amine monomers (precursors of M-NH_2_-20/21 with enhanced solubility), which undergo the hydrolysis reaction of Schiff-base (–N=CPh_2_) to amine (–NH_2_) prior to following Schiff-base condensation between –NH_2_ and ketone (Scheme 2c). Moreover, Mateo-Alonso and colleagues developed two 2D PPZs with semiconducting properties through polycondensation in the combination of M-NH_2_-16/M-CO-3 and M-NH_2_-28/M-CO-5, respectively.^73^ Besides, the enriched sp^2^-N makes 2D PPZs suitable cathodes in aqueous Zn-ion batteries, as illustrated in Zhu's work.^74^

### 3.4 2D poly(arylene vinylene)s (2D PAVs) by various polycondensation reactions

**Scheme 3:**
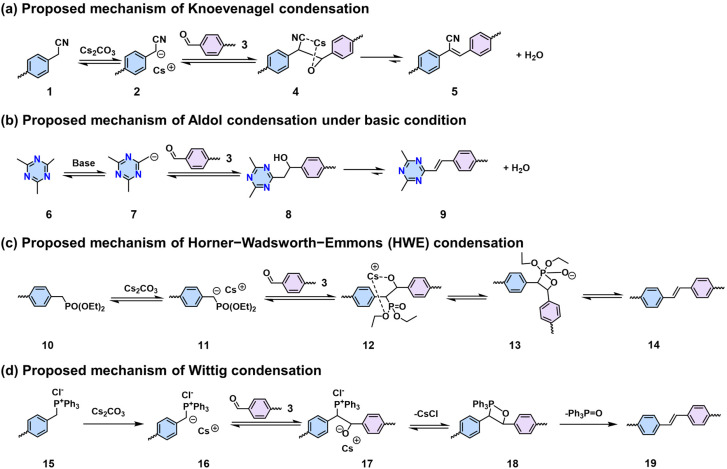
Methodologies and proposed mechanisms of (a) Knoevenagel, (b) Aldol-type, (c) HWE, and (d) Wittig condensations that can construct 2D PAVs.

In comparison to 2D PIs and 2D PPZs, 2D PAVs with robust polymeric skeletons have demonstrated inherently improved chemical stability and 2D π-conjugation, which have attracted significant interest over the last decade. Nevertheless, the synthesis of highly crystalline 2D PAVs remains challenging, given that the formation of vinylene linkages is semi-reversible, in contrast to the well-established Schiff-base chemistry with high reversibility. The current focus of 2D PAV development is to establish methodologies and design novel building blocks that facilitate the formation of highly crystalline structures. To date, a number of synthetic methodologies such as Knoevenagel,^18,75–77^ Aldol-type,^78–82^ HWE,^83^ and Wittig^84^ polycondensations have been developed for this class of materials (Scheme 3).

#### 3.4.1 Knoevenagel 2D polycondensation

**Scheme 4:**
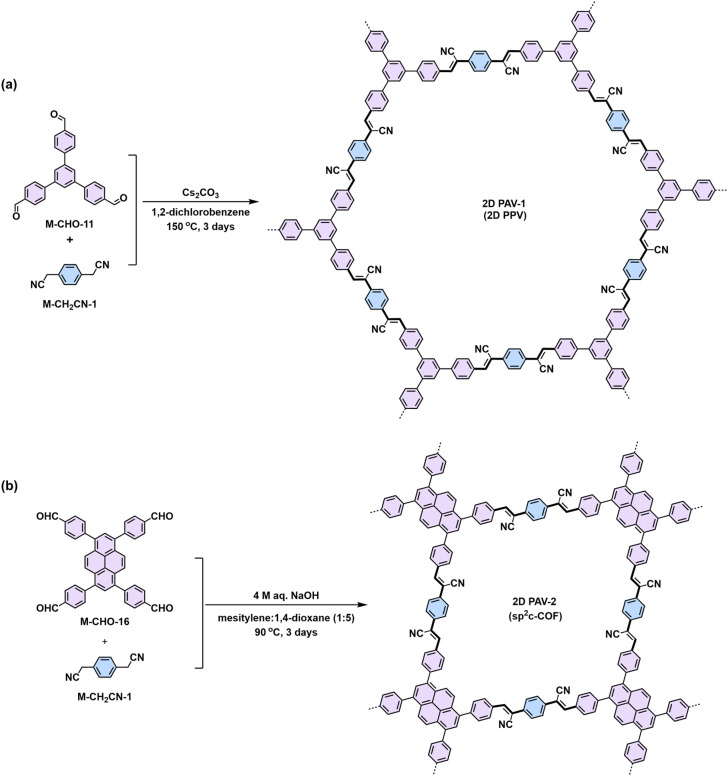
Synthesis of (a) 2D PAV-1 (2D PPV) with hexagonal lattice and (b) 2D PAV-2 (sp^2^c-COF) with 2D square lattice.

Knoevenagel condensation is a powerful tool for the construction of 2D PAVs. In a Knoevenagel reaction, the α-carbon atom of the aromatic methylene nitrile (1) can be easily converted to a reactive intermediate carbanion (2) under basic conditions and further stabilized through the conjugation effect. This carbanion will then nucleophilically attack the carbonyl group of (3) to form a C–C bond-linked intermediate (4). Finally, the elimination reaction releases a water molecule to establish the C=C bond (5) simultaneously (Scheme 3a). In 2016, our group reported the first highly crystalline cyano-vinylene-linked 2D poly(phenylene vinylene) (2D PPV, named as 2D PAV-1 in this Review) based on *p*-phenylenediacetonitrile (M-CH_2_CN-1) and 1,3,5-tris(4-formylphenyl)benzene (M-CHO-12) (Scheme 4a).^18^ The formation of cyano-vinylene linkages was identified by solid-state ^13^C-NMR and infrared spectroscopy. The exact structure of 2D PAV-1 is proposed to adopt a serrated stacking mode with neighboring layers slipped by 1/4 of the unit cell distance on average, and a distance of 0.35 nm separates the layers. The catalyst plays an important role in determining the reversibility of the vinylene linkages formation, and only the Cs^+^ ion can stabilize the carbanion intermediate (4), endowing quasi-reversible C–C bond formation and thus promoting the formation of a highly crystalline structure through a quasi-reversible self-healing process (Scheme 3a).^85^ At the same time, Jiang and colleagues reported 2D PAV-2 (sp^2^-c-COF) synthesized by Knoevenagel polycondensation between M-CH_2_CN-1 and 4,4′,4′′,4′′′-(pyrene-1,3,6,8-tetrayl)tetrabenzaldehydepyrene (M-CHO-16) (Scheme 4b).^75^ The lattice parameters for 2D PAV-2 are *a* = 34.4632 Å, *b* = 35.4951 Å, *c* = 3.7199 Å, *α* = *γ* = 90°, and *β* = 104.0277°, with a layer spacing of 3.58 Å in the *z*-direction. Doping with iodine, high-density radicals can be generated in 2D PAV-2, leading to a largely improved electrical conductivity from 6.1 × 10^−14^ S m^−1^ to 7.1 × 10^−2^ S m^−1^.

**Fig. 5:**
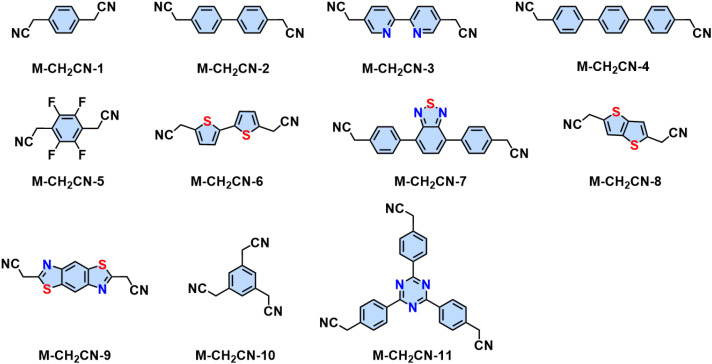
Representative aromatic methylene nitrile for the synthesis of 2D PAVs utilizing Knoevenagel polycondensation.

In the last several years, several aromatic aldehyde monomers and acetonitrile with different geometries have been successfully implemented in the synthesis of crystalline cyano-substituted 2D PAVs (Fig. 3 and 5), such as *C*_2_-symmetrical 4,8-bis(5-formyl-4-hexylthiophen-2-yl)benzo[1,2-*b*:4,5-*b*′]dithiophene-2,6-dicarbaldehyde (M-CHO-22),^86^*C*_3_-symmetrical 4,4′,4′′-(1,3,5-triazine-2,4,6-triyl)tribenzaldehyde (M-CHO-14),^87^ benzo[1,2-*b*:3,4-*b*′:5,6-*b*′′]trithiophene-2,5,8-tricarbaldehyde (M-CHO-24),^88^ and 2,3,8,9,14,15-hexa(4-formylphenyl)diquinoxalino[2,3-*a*:2′,3′-*c*]phenazine (M-CHO-27),^89^*C*_4_-symmetrical 4,4′,4′′,4′′′-(porphyrin-5,10,15,20-tetrayl)tetrabenzaldehyde (M-CHO-15),^76^ and 4,4′,4′′,4′′′-(perylene-2,5,8,11-tetrayl)tetrabenzaldehyde (M-CHO-17),^90^ as well as *C*_2_-symmetrical 2,2′-bipyridine-based 5,5-bis(cyanomethyl)-2,2-bipyridine (M-CH_2_CN-3)^91^ and 2,2′([2,2′-bithiophene]-5,5′-diyl)diacetonitrile (M-CH_2_CN-6),^86^ and 2,2′-(benzo[1,2-*d*:4,5-*d*′]bis(thiazole)-2,6-diyl)diacetonitrile (M-CH_2_CN-9).^77^

Highly crystalline cyano-substituted 2D PAVs can be synthesized by combining Knoevenagel polycondensation and water-assisted Michael addition–elimination.^92^ Through *in situ* high-temperature NMR measurements of model reactions and intermediate, we disclosed that Michael-addition–elimination is an efficient dynamic covalent chemistry for the direct C=C bond exchange, which endows the self-correction property to synthesize complex structures. The deep understanding of the reaction mechanism further guides the synthesis of four highly crystalline 2D PAVs with crystalline domain sizes ranging from 20 to 100 nm. Jiang proposed synthesizing crystalline 2D PAVs using a topology-templated synthesis strategy.^93^ In this strategy, the corresponding imine-linked 2D PIs were used as templates to facilitate and confine the growth of the cyano-substituted 2D PAVs to the *x*–*y* plane. Afterward, crystalline cyano-substituted 2D PAVs were obtained after hydrolyzing the corresponding 2D PI template. This template-assisted strategy can also synthesize crystalline 2D PAVs that are inaccessible through direct polymerization. Moreover, Hecht and colleagues demonstrated the on-surface synthesis of a single-layer 2D PAV through dynamic Knoevenagel polycondensation between 2,2′,2′′-((1,3,5-triazine-2,4,6-triyl)tris(benzene-4,1-diyl))triacetonitrile (M-CH_2_CN-11) and terephthalaldehyde. High-resolution STM characterizations reveal a covalently connected honeycomb lattice with domains up to 70 nm.^94^

**Scheme 5:**
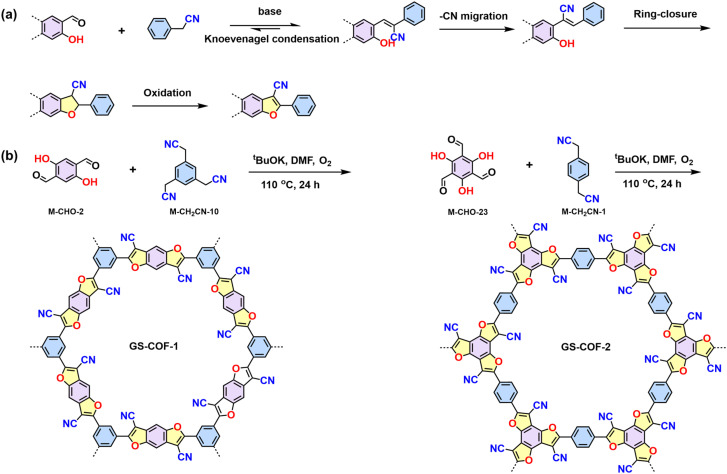
(a) Proposed mechanism of benzodifuran linkage formation. (b) Synthesis of GS-COF-1 and GS-COF-2.

Unlike the other 2D CPs constructed by the dynamic Knoevenagel polycondensation, Gu *et al.* reported the synthesis of crystalline 2D CPs (GS-COF-1 and GS-COF-2) by connecting hydroxybenzene-aldehyde and acetonitrile building blocks to form cyano-substituted benzofuran linkages.^95^ In the initial process, the Knoevenagel condensation reversibly generates the frameworks, whereas consecutive cyanide migration, ring-closure, and oxidation reactions yield an irreversible heteroaromatic cyano-substituted benzofuran linkage (Scheme 5).

**Scheme 6:**
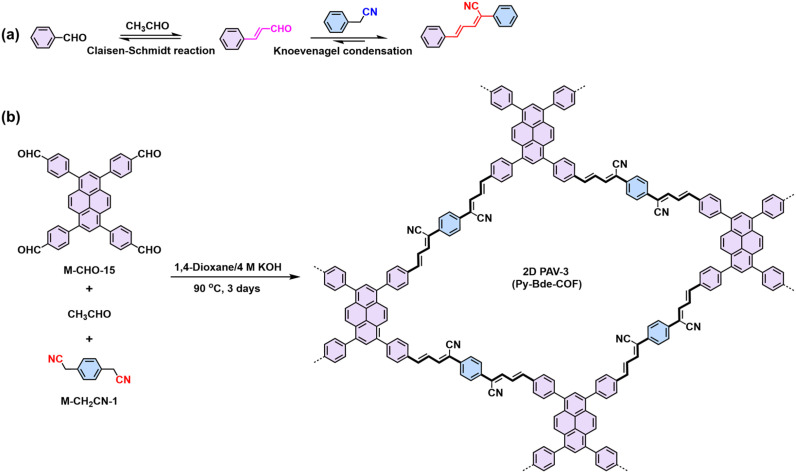
(a) Synthesis of the model compound using Knoevenagel condensation and Claisen–Schmidt reaction in one pot. (b) Synthesis of CN-equipped buta-1,3-diene-linked 2D PAV-3 (Py-Bde-COF).

Combining dynamic Knoevenagel condensation and Claisen–Schmidt reaction in one pot can build cyano-substituted buta-1,3-diene linked 2D PAVs, as illustrated by Gu's work.^96^ They initially synthesized a model compound with cyano-equipped buta-1,3-diene-linkage (Scheme 6a), which is almost coplanar with a slight torsion angle of 15.09° between one benzene ring and the buta-1,3-diene. The near-planar structure of buta-1,3-diene promotes the formation of layer-stacked 2D PAV-3 (Scheme 6b) condensed from consecutive Claisen–Schmidt reaction and Knoevenagel condensation.

**Scheme 7:**
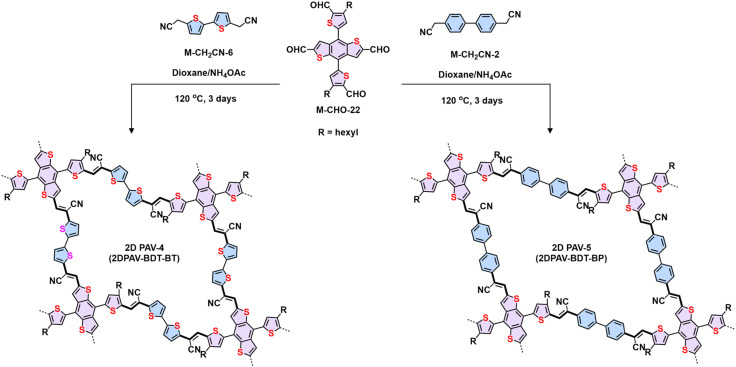
Synthesis of 2D PAV-4 (2DPAV-BDT-BT) and 2D PAV-5 (2DPAV-BDT-BP).

Regarding electronic applications, 2D PAVs with excellent charge transporting properties are highly desirable. Considering the thiophene-enriched building blocks feature highly planar backbone and strengthened p-orbital interactions, our group demonstrated the first example of fully thiophene (thienyl-benzodithiophene, M-CHO-22)-based 2D PAVs of 2D PAV-4 (2DPAV-BDT-BT, BT = bithiophene) and 2D PAV-5 (2DPAV-BDT-BP, BP = biphenyl) *via* Knoevenagel polycondensation (Scheme 7).^86^ Compared to the biphenyl-bridged 2D PAV-5, the fully thiophene-based 2D PAV-4 exhibits enhanced planarity, π-delocalization with a reduced band gap (1.62 eV), and significant electronic band dispersion, as revealed by the optical absorption and density functional theory calculations of electronic band structures. Remarkably, temperature-dependent terahertz spectroscopy discloses a unique band-like transport and excellent room-temperature charge carrier mobility of 65 cm^2^ V^−1^ s^−1^ for the fully thiophene-based 2D PAV, exceeding the reported 2D CPs in powder form. Moreover, some acceptor units, including benzothiadiazole,^97^ benzobisthiazole,^98^ benzothiadiazole,^99^ 2,2′-bipyridine,^91^ 1,2,4-thiadiazole,^99^ and triazine^100^ have been incorporated into 2D PAVs for improved charge separation and reduced band gap.

The majority of 2D PAVs are obtained as polycrystalline powders by solvothermal synthesis, which exhibit poor processability, hindering their integration into devices. Our group prepared 2D PAV films using the SMAIS method *via* Knoevenagel polycondensation conducted under a three-step methodology: (i) formation of surfactant monolayer on the water surface; (ii) injection and adsorption of the aldehyde monomer under the surfactant monolayer; (iii) injection and adsorption of aromatic methylene nitrile monomer; (iv) subsequent 2D polymerization. The synthesized films have a large lateral size (∼28 cm^2^), tunable thickness (∼2–52 nm), and high chemical stability. They can be used as an anion-selective electrode coating for reversible and durable zinc-based dual-ion batteries.^39^ DFT calculations demonstrated that, in comparison to the synthesis in an aqueous solution, the on-water surface synthesis exhibits lower energy barriers for intermediates, indicating the considerable influence of the 2D confinement provided by the air/water interface, which effectively constrains the out-of-plane molecule movement.

#### 3.4.2 Aldol-type 2D polycondensation

In contrast to synthesizing cyano-substituted 2D PAVs using Knoevenagel 2D polycondensation, other Aldol-type (or extended Knoevenagel) polycondensation reactions enable the synthesis of unsubstituted-vinylene-linked 2D PAVs. As shown in Scheme 3b, a symmetric and electron-deficient methyl monomer (*e.g.*, 4,6-trimethyl-1,3,5-triazine, the compound 6) is essential for Aldol-type condensation, which can be readily deprotonated into a reactive carbanion species (7) by various bases (*e.g.*, NaOH, piperidine, dimethylamine). Then the aldehyde group of compound (3) is attacked by this nucleophilic intermediate, initiating a reaction that culminates in forming a C–C bond, yielding compound (8). Subsequent elimination generates water and unsubstituted vinylene-linked aromatic frameworks (9).

**Fig. 6:**
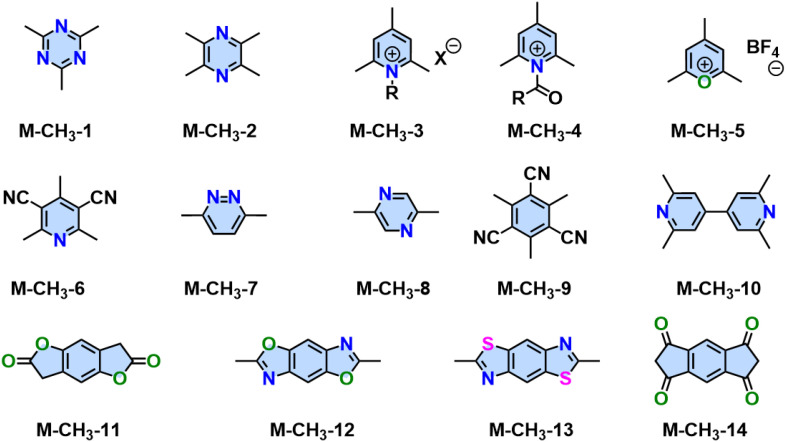
Representative aromatic monomers with active methyl groups for synthesizing 2D PAVs utilizing Aldol-type 2D polycondensation.

**Scheme 8:**
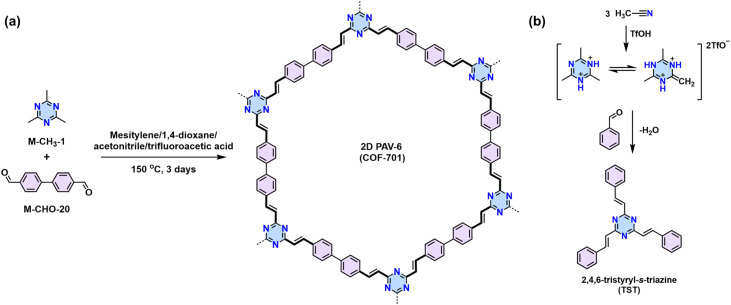
(a) Synthesis of the first un-substituted vinylene-linked 2D PAV-5 (COF-701). (b) Combination of cyclotrimerization and Aldol-type condensation to synthesize the model compound of 2,4,6-tristyryl-*s*-triazine (TST).

In 2019, Yaghi,^80^ Thomas,^78^ Perepichka,^82^ and Zhang^101^ reported the Aldol-type polycondensation method for the synthesis of 2D PAVs independently. For example, 2D PAV-6 (COF-701), was achieved by trifluoroacetic acid (TfOH)-catalyzed condensation between 2,4,6-trimethyl-1,3,5-triazine (M-CH_3_-1) and 4,4′-biphenyldicarbaldehyde (M-CHO-20) (Scheme 8a). Thomas and colleagues obtained two unsubstituted-vinylene linked 2D PAVs using base-catalyzed Aldol-type polycondensation between M-CH_3_-1 and terepthaldehyde (M-CHO-1)/1,3,5-tris(4-formylphenyl)benzene (M-CHO-11).^78^ Then, they combined cyclotrimerization of nitrile and Aldol-type condensation in one pot (Scheme 8b) and achieved crystalline 2D PAV-6 with a BET surface area of 736 m^2^ g^−1^.^80^ Other electron-deficient monomers, such as 2,4,6-trimethylpyridine derivatives (M-CH_3_-3 and M-CH_3_-4),^101–104^ 2,4,6-trimethylbenzene-1,3,5-tricarbonitrile (M-CH_3_-9),^105^ benzodifurandione (M-CH_3_-11),^106^ 2,6-dimethylbenzo[1,2-*d*:4,5-*d*′]bis(oxazole) (M-CH_3_-12),^97^ and 2,6-dimethylbenzo[1,2-*d*:4,5-*d*′]bis(thiazole) (M-CH_3_-13),^98^ and *s*-indacene-1,3,5,7(2*H*,6*H*)-tetraone (M-CH_3_-14)^107^ have also been successfully used for the construction of crystalline 2D PAVs (Fig. 6).

Aldol-type polycondensation also represents a powerful strategy to synthesize 2D PAVs on a large scale or directly as a thin film on substrates. In 2021, Zhang and colleagues developed a solvent-free method to synthesize 2D PAVs using benzoic anhydride as a catalyst.^79^ Later, Zhang *et al.* developed a simple and robust interfacial methodology for fabricating 2D PAV films on various solid substrates (*e.g.*, fluorine-doped tin oxide, aluminum sheet, polyacrylonitrile membrane).^108^ The resultant 2D PAV films show lateral sizes up to 120 cm^2^ and tunable thickness from tens of nanometers to a few micrometers.

#### 3.4.3 HWE and Wittig 2D polycondensation

**Scheme 9:**
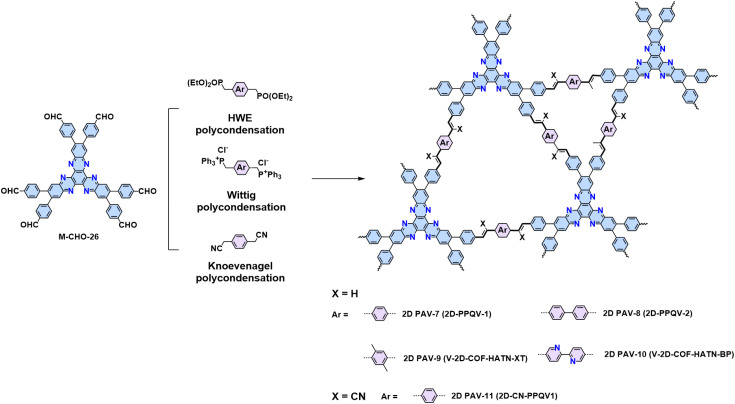
Synthesis of 2D PAV-7 (2D-PPQV-1), 2D PAV-8 (2D-PPQV-2), 2D PAV-9 (V-2D-COF-HATN-XT), and 2D PAV-10 (V-2D-COF-HATN-BP) through HWE or Wittig polymerization, together with 2D PAV-11 synthesized by Knoevenagel polycondensation.

Another methodology for synthesizing 2D PAVs is the HWE polycondensation as demonstrated by our group.^109^ The HWE-based 2D polymerization between aromatic phosphonate and aldehyde represents a more robust way to synthesize unsubstituted 2D PAVs with different topologies. The mechanism of the HWE reaction is proposed to involve three steps and a stabilized six-membered cyclic transition state with Cs^+^ (12), which includes an exclusive *trans*-vinylene bond (14) after the elimination step (Scheme 3c). Moreover, DFT simulations suggest that C–C single-bond formation is reversible (from 11 and 3 to 13), which is crucial to the formation of crystalline 2D PAVs (2D PAV-7 and 2D PAV-8, Scheme 9). Compared to cyano-vinylene-linked 2D PAV-11 developed by Knoevenagel polycondensation, 2D PAV-7 exhibits a narrower band gap (2.20 eV *vs.* 2.39 eV) and a bathochromic shift in photoluminescence of *ca.* 65 nm, indicative of an enhanced 2D conjugation in the latter. Besides the formation of crystalline 2D PAVs mentioned above, an in-depth exploration of the mechanism is needed to expand the monomer scope suitable for HWE polycondensation.

Similarly, the Wittig reaction from phosphonium ylides and aryl aldehydes has proved to be a useful strategy for constructing crystalline 2D PAVs (Scheme 3d).^84^ The (*trans*/*cis*-) *E*/*Z* selectivity of aryl–ylide-based Wittig reactions is generally poor. Our group tailored the high *E* selectivity through (I) elevating temperature as an additional energy source to provide the thermodynamic driving force; (II) screening proper catalyst to obtain more stable intermediates with preferred geometry and associated facial selectivity during C=C bond formation.^84^ Four different 2D PAVs are successfully synthesized by combining 2,3,8,9,14,15-hexa(4-formylphenyl)diquinoxalino[2,3-*a*:2′,3′-*c*]phenazine (M-CHO-26) and four ylides (Scheme 9). The resultant 2D PAVs (2D PAV-7, 2D PAV-8, 2D PAV-9, and 2D PAV-10) present crystalline dual-pore structures and robust unsubstituted vinylene linkages with high chemical stability. THz spectroscopy reveals a charge carrier mobility of 10.3 cm^2^ V^−1^ s^−1^ for 2D PAV-9 at room temperature.

### 3.5 2D polyarylenes (2D PAs) by aryl–aryl coupling

**Fig. 7:**
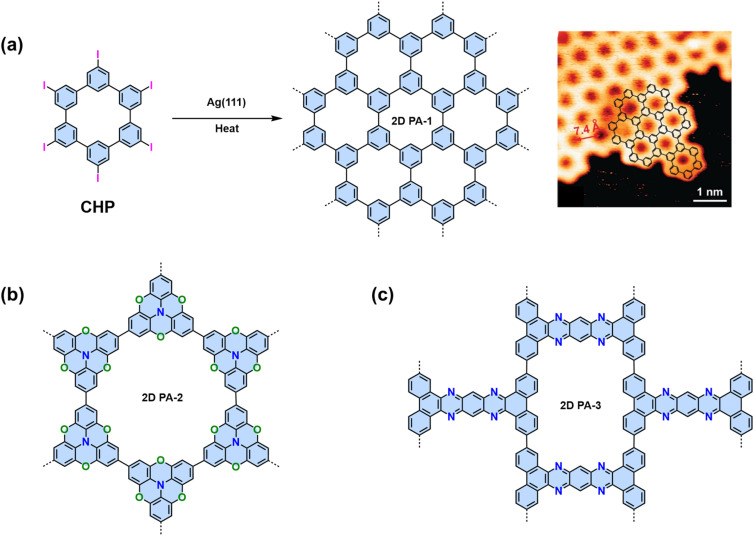
(a) On-surface synthesis of 2D polyphenylene (2D PA-1) and the corresponding STM image. (b) Chemical structure of 2D PA-2 synthesized on Au(111) surface. (c) Chemical structure of 2D PA-3 synthesized by solid-state synthesis.

Considering the different linking chemistries developed so far, the direct connection of aromatic moieties is advantageous to establish π-conjugation for efficient charge transfer. The synthesis of crystalline 2D CPs linked by C–C bonds has been considered one of the holy grails of synthetic chemistry.^16,110,111^ Both Au(111) and Ag(111) surfaces can promote aryl–aryl coupling. In 2009, our group and cooperation partner specifically designed a hexaiodo-substituted macrocycle cyclo-hexa-*m*-phenylene (CHP), which adopts an essentially planar conformation on the Ag(111) surface.^17^ Annealing to temperatures above 570 K initiates the coupling reaction. Six-fold aryl–aryl coupling of the CHP macrocycles results in a monolayer 2D polyphenylene (2D PA-1) with a 2D honeycomb network with a domain size up to 50 nm (Fig. 7a). The irreversible C–C bond formation prevents self-correction, which leads to many defects, *i.e.*, 1D chains will be introduced into the expected 2D networks.

In order to address the high defect density issue, Contini and coworkers realized mesoscale ordered 2D PAs by dosing rigid achiral heterotriangulene molecules (tribromotrioxaazatriangulene, TBTANG) on hot (210 °C) Au(111) surfaces (Fig. 7b). This process minimizes the formation of network defects and favors the growth of highly extended (>100 nm) hexagonal structures.^112^ The ordered and defect-less mesoscale structure facilitated the use of angle-resolved photoelectron spectroscopy for analyzing the 2D polymer. This technique disclosed the presence of both a Dirac cone and flat bands within their kagome lattices, rendering this 2D PA a fascinating arena for exploring phenomena such as the anomalous Hall effect, surface superconductivity, and superfluid transport.

In addition the on-surface synthesis on a metal substrate, in 2017, Loh and coworkers reported the solid-state synthesis of another 2D PA based on 2,7,13,18-tetrabromodibenzo[*a*,*c*]dibenzo[5,6:7,8]quinoxalino-[2,3-*i*]phenazine (2-TBQP) (Fig. 7c). Firstly, 2-TBQP was sublimated to generate needle-like single crystals of monomers with a zigzag and tightly packed lamellar structure through a displaced face-to-face π–π interaction at a distance of 3.269 Å.^113^ After holding the temperature at 520 °C for six hours, the monomer single crystals transformed into a shiny grey needle-shaped polymer. Scanning electron microscopy studies revealed that the bulk precursor 2-TBQP crystal underwent a transformation into polymers with apparent lamellar features. The embedded polymeric sheets are aligned parallel to the *c*-axis of the original precursor 2-TBQP crystal. The use of Scotch tape facilitates the exfoliation of these crystals into micrometer-sized, nanometer-thick sheets.

**Fig. 8:**
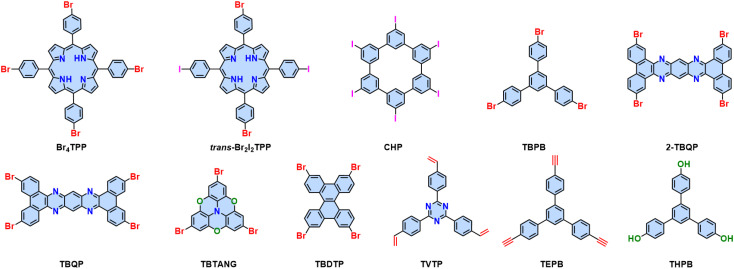
Building blocks investigated for the formation of 2D CPs on metal surfaces.

It should be noted that aryl–aryl coupling reactions are not limited to the Ullmann-type coupling. For example, monomers with terminal alkenyl or alkynyl groups, *e.g.*, 2,4,6-tris(4-vinylphenyl)-1,3,5-triazine (TVTP),^114^ and 1,3,5-tris-(4-ethynylphenyl)benzene (TEPB),^114^ can trigger a cyclotrimerization towards the synthesis of 2D PAs on the gold surface. In addition, a 1,3,5-tris(4hydroxyphenyl)benzene (THPB)^115^ monomer can undergo an oxidative C–C homocoupling into 2D PA followed by a reductive cleavage of the hydroxyl groups on both the Au(111) and Ag(111) surfaces (Fig. 8).

### 3.6 2D poly(benzimidazobenzophenanthroline)s (2D BBLs)

**Scheme 10:**
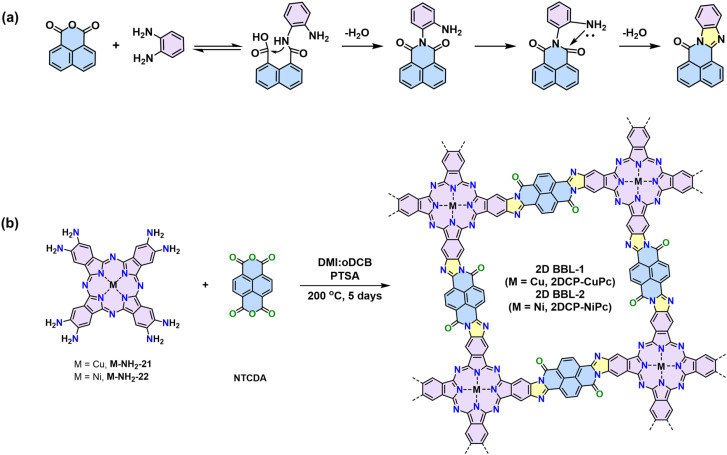
(a) Proposed reaction mechanism of imidazole linkages. (b) Synthesis of the two phthalocyanine-based BBL-ladder-type crystalline 2D CPs.

Ladder-type CPs, which consists of linearly fused aromatic subunits, are highly desirable due to the rigidity of the polymer backbone and the enhanced π-orbital overlapping. Charge delocalization is significantly facilitated because of the reduced conformational and energetic disorder endowed by the rigid “lock-in” backbone conformation.^116^ Theoretical calculations on various phthalocyanine-based model compounds and 1D CPs suggest a superior conjugation degree (*i.e.*, an inferior highest occupied molecular orbital–lowest unoccupied molecular orbital gap) for the BBL-ladder-type structure to those model compounds/polymers linked by imines, pyrazines, vinylenes, *etc*. The formation of BBL-imidazole linkage involves two consecutive steps. The first step is the quasi-reversible formation of imide-intermediate, followed by irreversible intramolecular dehydration (Scheme 10a). However, due to the harsh conditions required for the imidazole ring formation from *o*-diamine and anhydride (*e.g.*, temperatures more than 200 °C), the synthesis of crystalline 2D BBL has been a great challenge.^116^

Our group reported the first crystalline 2D BBLs based on phthalocyanine building block.^4^ pXRD analysis reveals the crystalline nature with distinct diffractions from different crystallographic planes, corresponding to a slipped-AA stacking geometry. We disclosed that *p*-toluenesulfonic acid can improve the reactivity due to its unique protonation–deprotonation ability. Crystalline 2D BBL-1 and 2D BBL-2 were synthesized by polycondensation with PTSA as the catalyst (Scheme 10b). The achieved 2D BBL-1 and 2D BBL-2 show highly delocalized π-electrons and strongly dispersive electronic bands. They present narrow optical bandgaps of 1.3 eV and a unique band transport feature with high charge mobility of ∼970 cm^2^ V^−1^ s^−1^ at room temperature and ∼1390 cm^2^ V^−1^ s^−1^ at 78 K, demonstrating the great potential of 2D CPs in high-performance organic electronics. This condition can be generalized to the synthesis of other 2D BBLs, *e.g.*, polycondensation between triphenylene-based M-NH_2_-19 and naphthalene tetracarboxylic dianhydride leads to another crystalline 2D BBL, which can be used for ultrafast electrochemical proton storage.^40^

## 4 Conclusions and outlook

Over the last decade, we have witnessed the rapid development of 2D CPs, not only as a new class of porous materials with long-range ordered structures but also as a playground for integrating pre-designed electronic and quantum functionalities. This review highlights the importance of synthetic methodologies to fully unlock the potential of 2D CPs through various linkage chemistry and the structural/topological designs. We commence with a summary of the most popular 2D PIs, outlining the efforts of the synthetic community in relation to Schiff-base chemistry. These efforts are directed towards enhancing conjugation and chemical stability, *i.e.*, 2D PI derivatives by post-modification or multicomponent one-pot synthesis, and ladder-type 2D PPZs by consecutive Schiff-base reactions.

More importantly, 2D PAVs have experienced substantial advancement in the last few years, with the development of synthetic chemistry and the investigation of their physical properties. A variety of synthetic methodologies, including Knoevenagel, Aldol-type, HWE, and Wittig 2D polycondensation reactions, have been established for 2D PAVs with enlarged crystal domains. The replacement of C=N linkages in 2D PIs with C=C linkages in 2D PAVs not only enhances the stability by protecting it from nucleophile attack but also improves the conjugation and delocalization of the electron cloud by reducing the polarity of the linkages. To date, the synthesis of 2D PAs has predominantly involved the formation of C–C bonds on the metal surface. This unique surface chemistry can overcome the irreversibility of C–C coupling in traditional wet chemistry. However, the obtained thin layers usually show limited domain size and are unsuitable for further device integration. It is evident that new strategies, such as new monomer design, are still required to overcome the limitations associated with the polymer domain size. Finally, we illustrate the development of ladder-type 2D BBLs with unique band-like transport and exceptionally high charge carrier mobility, a “booster dose” to this field. Notwithstanding the considerable advances that have been made, a number of challenges remain to be addressed.

(1) As illustrated, the library of 2D CPs has considerably expanded with the development of novel building blocks and synthetic methodologies. Nonetheless, the design of 2D CPs to achieve charge mobility higher than 10 cm^2^ V^−1^ s^−1^ at the device level remains to be demonstrated. Molecular design strategies to facilitate π-delocalization, narrow band gap, and improve charge mobility are highly demanding. Novel planar conjugated building blocks, new conjugated linkages, and unique 2D lattice and stack modes are always welcome. Moreover, the donor–acceptor molecular design in 1D CPs can be adapted to develop the electron-deficient n-type 2D CPs.

(2) While narrow optical band gap and high charge carrier mobility are at reach by rationally tuning the structures of 2D PAVs and 2D BBLs, the role of intrinsic and extrinsic (*e.g.*, boundaries and defects) contributions remains to be elucidated. It is imperative that the development of highly crystalline or single-crystalline 2D PAVs/BBLs be pursued before any attempt is made to establish structure–property relationships that are currently ambiguous. A more profound comprehension of the underlying reaction mechanisms is of paramount importance in order to regulate the reactivity and reversibility of monomers, which, in turn, is fundamental to the process of crystal growth. Before this objective can be achieved, it is essential to ascertain whether the crystallization of 2D CPs is thermodynamically or dynamically controlled. The deployment of *in situ* and *ex situ* characterization techniques, encompassing both spectroscopic and crystallographic analyses, will prove invaluable in elucidating the underlying processes occurring during crystallization.

(3) Solvothermal synthesis is a predominant approach for producing 2D CPs, yet they offer limited control over crystal growth, and the bulk samples obtained present challenges in terms of processing and integration into devices. The quality of exfoliated bulk crystals into nanosheets is still unsatisfactory, which relies on the development of 2D CPs with large crystal sizes and weak interlayer coupling, thus facilitating the exfoliation of 2D CP layers. Developing large-area, free-standing 2D CP films through wet interface-assisted strategies is beneficial for easy device integration. However, these interfacial strategies have been limited thus far, with successful syntheses heavily dependent on the suitable solubility of certain building blocks in water or organic solvents. In light of these considerations, the synthesis of processable 2D CPs and their integration into functional devices, *e.g.*, field-effect transistors and bioelectronics, is urgently needed to drive future advancements in the field.

## Data availability

No primary research results, software or code have been included and no new data were generated or analysed as part of this review.

## Conflicts of interest

There are no conflicts to declare.
